# Potential of *Herbaspirillum* and *Azospirillum* Consortium to Promote Growth of Perennial Ryegrass under Water Deficit

**DOI:** 10.3390/microorganisms9010091

**Published:** 2021-01-01

**Authors:** Sandra Cortés-Patiño, Christian Vargas, Fagua Álvarez-Flórez, Ruth Bonilla, German Estrada-Bonilla

**Affiliations:** 1 Corporación Colombiana de Investigación Agropecuaria (AGROSAVIA), C.I. Tibaitatá, Km 14 Via Mosquera-Bogotá, Mosquera, Cundinamarca 250047, Colombia; scortesp@agrosavia.co (S.C.-P.); rbonilla@agrosavia.co (R.B.); 2 Departamento de Biología, Facultad de Ciencias, Universidad Nacional de Colombia, Bogotá 111321, Colombia; cdv22_@outlook.com (C.V.); falvarez@unal.edu.co (F.Á.-F.)

**Keywords:** endophytic bacteria, growth-promoting trait, drought, *Lolium*

## Abstract

Plant growth-promoting bacteria (PGPB) can mitigate the effect of abiotic stresses on plant growth and development; however, the degree of plant response is host-specific. The present study aimed to assess the growth-promoting effect of *Herbaspirillum* (AP21, AP02), *Azospirillum* (D7), and *Pseudomonas* (N7) strains (single and co-inoculated) in perennial ryegrass plants subjected to drought. The plants were grown under controlled conditions and subjected to water deficit for 10 days. A significant increase of approximately 30% in dry biomass production was observed using three co-inoculation combinations (*p* < 0.01). Genomic analysis enabled the detection of representative genes associated with plant colonization and growth promotion. In vitro tests revealed that all the strains could produce indolic compounds and exopolysaccharides and suggested that they could promote plant growth via volatile organic compounds. Co-inoculations mostly decreased the in vitro-tested growth-promoting traits; however, the co-inoculation of *Herbaspirillum* sp. AP21 and *Azospirillum brasilense* D7 resulted in the highest indolic compound production (*p* < 0.05). Although the *Azospirillum* strain showed the highest potential in the in vitro and in silico tests, the plants responded better when PGPB were co-inoculated, demonstrating the importance of integrating in silico, in vitro, and in vivo assessment results when selecting PGPB to mitigate drought stress.

## 1. Introduction

Plant growth-promoting bacteria (PGPB) are beneficial microorganisms that can improve nutrient acquisition, increase yield, and improve the resistance of plants to biotic and abiotic stresses [1]. They have become a crucial component of sustainable agriculture because their use can help reduce the environmental problems associated with intensive crop production, during which high doses of agrochemicals are needed for fertilization and pest control. However, the efficacy of PGPB depends on the interaction of several factors, including soil, plant, and microbial interactions, and this has posed critical barriers to their widespread use [2]. To increase the efficacy of PGPB, some commercial products contain engineered microbial consortia; in other words, different bacterial strains co-exist in the inoculant, thereby having the potential of being more effective than a single strain [3].

Normally, in the case of a consortium, the production and formulation stages are more difficult. However, the use of more than one strain may ensure that at least one of the strains will succeed in colonizing the rhizosphere, or the plant tissue if the strains are endophytic [4]. Endophytic bacteria are defined as bacteria that are able to colonize and live inside plant tissues. After colonizing the rhizosphere, these bacteria can live inside the roots, stems, and leaves of plants without harming their host [5,6]. Many of these bacteria are facultative colonizers, i.e., they have a metabolic battery that allows them to survive and be competitive in the rhizosphere, to colonize the plant tissue, and to actively communicate with the surrounding environment inside their host [7]. Inside the plant, these bacteria promote growth by producing phytohormones (auxins, gibberellins, and abscisic acid), improving nutrient acquisition (N, P, and Fe), promoting the activity of the enzyme 1-aminocyclopropane-1-carboxylate (ACC) deaminase (which helps reduce dangerous ethylene levels), and protecting against pathogens (via induced systemic resistance (ISR)) [5,8]. Facultative endophytic bacteria can also improve plant growth-promoting (PGP) traits in other bacteria. Puente et al. [9] found that the foliar inoculation of *Azospirillum brasilense* Az39 increased the number and biomass of root nodules produced by *Bradyrhizobium japonicum* E.109 in soybean plants. This positive interaction has mainly been observed between *Azospirillum* and nodule-forming rhizobia [10,11] and has been reported to improve the response of soybean plants to drought [12] and of maize plants to salinity stress [13].

*Azospirillum* species are the most studied PGPB, mainly because of their ability to produce growth regulator compounds, such as indole-3-acetic acid (IAA) and gibberellins, and to exert growth-promoting effects in a diverse set of hosts [14,15]. This genus was first isolated because of its ability to fix nitrogen in gramineous plants [16], similar to *Herbaspirillum*, another endophytic genus that has been shown to promote growth, mainly in sugarcane [17], rice [18], maize [19], and red clover [20] plants. Bacteria belonging to the genus *Pseudomonas* have mainly been used for biocontrol [21]; however, more recently, the presence of the ACC deaminase enzyme has been shown to confer growth-promoting traits and protective in plants in response to abiotic stresses, such as salinity [22] and drought [23].

Perennial ryegrass (*Lolium perenne* L.) is commonly used for livestock feeding. Although it has better nutritional qualities than other grasses, it is sensitive to water deficit, mainly because of its shallow root system [24]. Given the increasing use of this grass in Colombia, sustainable strategies are needed to reduce the effect of water deficit on its growth and biomass production. PGPB could help improve plant growth under this condition. Therefore, the present study aimed to assess the potential growth-promoting effect of the co-inoculation of four *Azospirillum, Herbaspirillum*, and *Pseudomonas* strains in perennial ryegrass under water deficit and to explore the in silico and in vitro potential of these strains in order to understand how some of their multiple PGP mechanisms could synergistically act to promote plant growth.

## 2. Materials and Methods 

### 2.1. Bacterial Strains

The bacterial strains used in the present study are part of the germplasm collection of the Colombian Corporation for Agricultural Research, Agrosavia. The strains were previously identified on the basis of partial sequencing of the 16S rRNA gene and deposited in GeneBank under the following accession numbers: MT302839 (*Azospirillum* sp. D7), MT302837 (*Pseudomonas* sp. N7), MT302838 (*Herbaspirillum* sp. AP02), and MT302840 (*Herbaspirillum* sp. AP21). The strains are hereafter referred to as D7, N7, AP02, and AP21, respectively, in this section to describe the treatments. The compatibility of the strains was tested according to the methodology of Loaces et al. [25], and no inhibitory effect was observed in the solid medium.

For all the assays, bacterial cells were obtained from pure cultures in solid DYGS medium [26] and then grown in liquid broth for 24 h at 30 °C and 120 rpm. After incubation, the bacterial concentration was standardized to optical density (OD)_600nm_ = 0.5. (~10^8^ colony forming units (CFU) mL^−1^. The bacterial strains for co-inoculations were mixed in a 1:1 ratio (*v/v*), to obtain 5 mL of mixed inoculants, right before plant inoculation. For the uninoculated treatments, sterile liquid medium was used.

### 2.2. Genome Sequencing, Phylogenomic Analysis, and Annotation

Phylogenomic analysis was performed to obtain more information about the taxonomy of the strains. In brief, genomic DNA of the four strains was extracted using the QIAamp DNA Mini Kit (Qiagen, CA, USA). The DNA quality was assessed with a Qubit fluorometer (Thermo Fisher Scientific, Waltham, MA, USA) using the Quant-iT dsDNA HS Assay Kit (commercial protocol, Thermo Fisher Scientific, Waltham, MA, USA). The genomic library was prepared using Nextera XT (Illumina, San Diego, CA, USA), and sequencing was performed using MiSeq (Illumina) with 250 bp pair-end reads at the Microbial Genomics Laboratory at Universidad El Bosque, Bogotá, Colombia. The quality of the raw data was analyzed using FastQC [27], and low-quality reads were removed using ILLUMINACLIP, a tool in Trimmomatic v0.32 [28]. High-quality reads were assembled into contigs using IDBA-UD [29]. Taxonomic mapping was performed using MyTaxa [30]. The complete genomes of the strains are available at the NCBI genome database under the following accessions numbers: AP02: SAMN16830020; D7: SAMN16830199; N7: SAMN16830036; AP21: SAMN15498633.

To compute the phylogeny of the strains, the method of Yeoman et al. [31] was used. In brief, the complete genomes of *Azospirillum*, *Herpaspirillum*, and *P. fluorescens* strains stored in NCBI were identified and downloaded. Then, single copy genes of the strains were aligned and converted to amino acid sequences. Gaps were eliminated using trimAI v1.4.rev22 [32]. IQ-TREE v1.5.5 was then used, and a maximum likelihood tree was constructed with the general matrix model WAG [33].

In addition, representative genes associated with plant growth promotion were identified. A database was built with sequences of genes related to exopolysaccharide (EPS) and lipoprotein (LPS) production (*noeJ, noeL, rfbB*, *and rfbC*), biofilm formation (*flmA* and *flmB*), auxin synthesis (*iaaM* and *ipdC*), and volatile organic compound (VOC) production (*gacA* and *gacS*). In parallel, the coding sequence was estimated using Prodigal (Doug et al., 2010). Finally, the USEARCH global algorithm [34] was used to determine the presence of these genes in the genomes of the strains used in the present study.

### 2.3. PGP Effects of Single and Co-Inoculated PGPB in Perennial Ryegrass under Water Deficit

Seeds of perennial ryegrass var. One50 were surface-sterilized in 3% *v* *v*^−1^ hypochlorite and 70% *v* *v*^−1^ ethanol. Following this, the seeds were planted in gel nutritive medium [35] and incubated for 5 days at 25 °C in darkness. The germinated seeds were transplanted into pots containing 400 g of unsterile soil and inoculated with 5 mL of each bacterial inoculum. The soil used was classified as Typic Hapludand of the order andisol [36] with the following characteristics: pH 6.04; organic matter (130 g kg^−1^); effective coefficient for cationic exchange (28.39 cmol kg^−1^); P (381 mg kg^−1^); K (4.18 cmol kg^−1^); S (23.90 mg kg^−1^); Ca (15.95 cmol kg^−1^); Mg (8.78 cmol kg^−1^); Na (0.40 cmol kg^−1^); and Fe (620.03 mg kg^−1^). The treatments consisted of the four strains used individually (AP02, AP21, D7, and N7) and in dual co-inoculations (AP02 + AP21, AP02 + D7, AP02 + N7, AP21 + D7, AP21 + N7, and D7 + N7), in addition to stressed and watered uninoculated controls. The pots were placed in a growth room under controlled conditions (50% relative humidity (RH), 17–25 °C, 16/8 h photoperiod) and irrigated at field capacity for 21 days. Then, the irrigation was suspended for all the treatments, except for the watered control. The water was withheld for 10 days, following which the height, relative water content (RWC), and dry weight (DW) were measured. Nine pots per treatment were used in a completely randomized design.

The leaves of each plant were placed in an oven at 70 °C for 72 h and then weighed to determine their DW. RWC was measured according to the method described by Sade et al. [37] for leaves of gramineous plants. In brief, a 6 cm piece of the leaf blade (starting from the tip) was cut with scissors from a completely developed mature leaf from each plant. The leaves were placed in a previously weighed plastic bag, and their fresh weight (FW) was measured. Following this, 3 mL of a 5 mM CaCl_2_ solution was added to a bag that was then sealed and kept for 8 h at room temperature. Next, the excess water was removed with paper towels to measure the turgid weight (TW) of the leaves. Finally, as mentioned above, the leaves were placed in an oven at 70 °C for 72 h to measure their DW. RWC was calculated using the following equation: RWC = (FW – DW/TW – DW) × 100.

### 2.4. In Vitro PGP Traits of the Bacterial Co-Inoculation Combinations

The PGP activities of the three co-inoculation combinations selected in the previous assay were compared with those of the individual strains. The treatments for these experiments were as follows: (1) AP02, (2) AP21, (3) D7, (4) N7, (5) AP02 + AP21, (6) AP02 + N7, and (7) AP21 + D7.

#### 2.4.1. Indolic Compounds’ (ICs) Production

The production of this growth regulator was measured according to the method of Glickmann and Dessaux [38], with some modifications. In brief, 50 µL aliquots from each treatment (single or co-inoculated strains) were inoculated in flasks with 5 mL of DYGS broth supplemented with 5 mM of L-tryptophan. The flasks were incubated in an orbital shaker (150 rpm) at 30 °C for 72 h. Following this, 1 mL of the content was centrifuged at 17,390× *g* for 5 min. Next, 100 µL of the supernatant was added to ELISA microplates and mixed with 100 µL of Salkowski reagent. The microplate was kept in the dark for 30 min at room temperature, and the OD was measured at 535 nm. The results were compared to an IAA standard curve. Three flasks per treatment were used as replicates.

At the end of the incubation period, the bacterial population in each flask was measured by the serial dilution technique. Serial dilutions (from 10^2^ to 10^7^ CFU mL^−^^1^) were performed using a 0.85% *w* *v*^−1^ NaCl solution. Following this, 20 µL of each dilution was added to Petri dishes containing Congo red solid medium [39] and left to dry. The Petri dishes were incubated at 30 °C for 48 h, and the colonies were counted as CFU mL^−1^.

#### 2.4.2. EPS Production

EPS production was assessed using the method described by Moreno-Galván et al. [40]. In total, 100 µL of each bacterial inoculum was added to flasks containing 10 mL of NFb liquid medium [26] and incubated in an orbital shaker (150 rpm) at 30 °C for 96 h. The bacterial broth was then transferred to Falcon tubes, and 200 µL of 0.26 mg L^−1^ EDTA and 100 µL of 5 mg L^−1^ NaCl were added to it. The contents of the tubes were mixed, and the tubes were then centrifuged at 5220× *g* for 10 min. Following this, the supernatant was collected and 10 mL of cold ethanol (−80 °C) was added to it. After 24 h of incubation at 4 °C, the tubes were centrifuged under the above-mentioned conditions. The supernatant was discarded, and 5 mL of distilled water was added to the pellet. EPSs were quantified using the colorimetric method of Dubois et al. [41]. In brief, a 300 μL aliquot of each sample was mixed with 300 µL of 5 mg mL^−1^ phenol and 1.5 mL of concentrated H_2_SO_4_. The mixture was stabilized at 28 °C for 30 min in a water bath, following which the absorbance was read at 490 nm. Three flasks per treatment were used as replicates. The bacterial concentration in each flask was measured at the end of 96 h, as described above for IC measurements.

#### 2.4.3. VOC Production

VOC production by the strains was measured according to the in vitro method described by Park et al. [42], with some modifications. Six surface-sterilized seeds of perennial ryegrass were placed in one half of a divided plate containing gel nutritive medium [35]. A suspension of each single inoculation or co-inoculation (OD_600nm_ = 0.2) was added to the other half of the plate containing DYGS medium. Seedling germination, coleoptile length, and radicle length were assessed after 4 days of incubation in the dark at 25 °C. Four plates per treatment were used.

### 2.5. Plant Tissue Colonization in Synthetic Medium 

For each strain, bacterial broths were prepared as described above, centrifuged at 5220× *g* for 5 min, washed twice with sterile distilled water, and diluted to obtain a concentration of 10^6^ CFU mL^−1^. The suspensions were then left alone or mixed for co-inoculations (1:1 *v/v*). Previously surface-sterilized and germinated seeds of ryegrass perenne var. One50 were soaked in the bacterial suspensions and kept in an orbital shaker at 120 rpm at room temperature for 1 h. Following this, the seeds were washed with sterile distilled water to remove excess bacterial suspensions and added to plates containing solid nutritive medium [35]. The plates were incubated in a growth room (60% RH, 25 °C, 12/12 h photoperiod) for 7 days, and the seedlings were cut and prepared for the most probable number assay as follows.

The seedlings were surface sterilized with 3% *v* *v*^−1^ NaCl and 70% *v* *v*^−1^ ethanol for 1 and 2 min, respectively, and washed thrice with sterile distilled water. For each treatment, all the roots and shoots were mixed to obtain sufficient biomass for counting the number of endophytic diazotrophic bacterial cells. Serial dilutions were performed using a saline solution (0.85% *w* *v*^−1^) until the 10^−8^ dilution was reached. Following this, 100 µL of each dilution was added to a vial containing 4.5 mL of NFb semi-solid medium [26]. For each dilution, three vials were used. The vials were incubated for 5 days at 30 °C. Following this, the number of vials with a change in color and pellicle-like growth were counted for each dilution. The bacterial concentration (CFU mL^−1^) was determined according to the McCrady table [26].

### 2.6. Data Analysis

Data for the plant growth parameters under water deficit and the in vitro tests of the plant growth-promoting traits were assessed using the nonparametric Kruskal–Wallis H test followed by all pairwise comparison of the means (*p* < 0.05). All analyses were performed using R Studio version 4.0.2.4.

## 3. Results

### 3.1. Phylogenomic Analysis and Annotation of Genes Related to Plant–Microbe Interactions and PGP Traits

**Figure 1:**
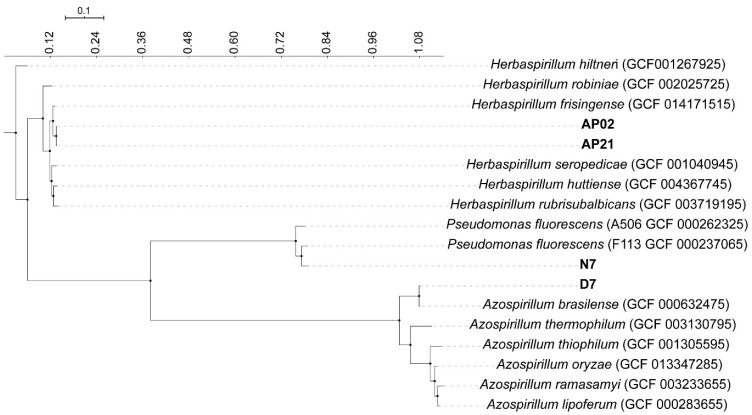
Phylogenetic relationships among the four strains (AP02, AP21, D7, and N7) used in this study and 14 reference strains available in NCBI. To compute this phylogeny, the workflow mentioned by Yeoman et al. (31) was used. Bar: tree scale.

To provide more accurate information about the phylogeny of the strains used in the present study, we assessed their evolutionary relationship based on their complete genomes. The analysis included 14 reference strains available in NCBI. Maximum likelihood models revealed three clades associated with the three genera used in the study: *Azospirillum*, *Herbaspirillum*, and *Pseudomonas*. Although the genera of these strains were previously determined by partial sequencing of their 16S rRNA, this analysis allowed us to associate the strains N7 and D7 with *P. fluorescens* and *A. brasilense*, respectively (*p* < 0.05) (Figure 1). In addition, when integrating these results with those of MyTaxa, we noticed that the strains AP02 and AP21 possibly belonged to a different *Herbaspirillum* species than those for which the genomic data were available (*p* > 0.05); this finding should be confirmed by molecular and physiologic analysis.

The search for genes related to plant–microbe interactions and PGP traits provided some insights about the potential of these strains. Genes related to IAA production, EPS production, and biofilm formation were found in *A. brasilense* D7 (Table 1). Moreover, *acdS*, related to the activity of the ACC deaminase enzyme, was present in the *Herbaspirillum* strains AP02 and AP21 and the *Pseudomonas* strain N7, confirming the enzymatic activity observed in previous in vitro assays. The *Pseudomonas* strain N7 contained *iaaM*, related to the indole-3-acetamide (IAM) pathway for IAA production, while *A. brasilense* D7 and *Herbaspirillum* sp. AP02 contained *ipdC*, related to indole-3-pyruvic acid production. The genes *rfbB* and *rfbC*, reported as necessary for plant colonization in *H. seropedicae* [43], were not detected in the *Herbaspirillum* strains AP02 and AP21; however, protein sequences related to these genes were detected in the *Azospirillum* strain D7 and the *Pseudomonas* strain N7. Finally, *gacA* and *gacS*, reported to regulate the ability of *P. fluorescens* to produce VOCs, were detected in the *Pseudomonas* strain N7.

### 3.2. Plant Growth Promotion by Single and Co-Inoculated PGPB under Controlled Conditions

**Figure 2:**
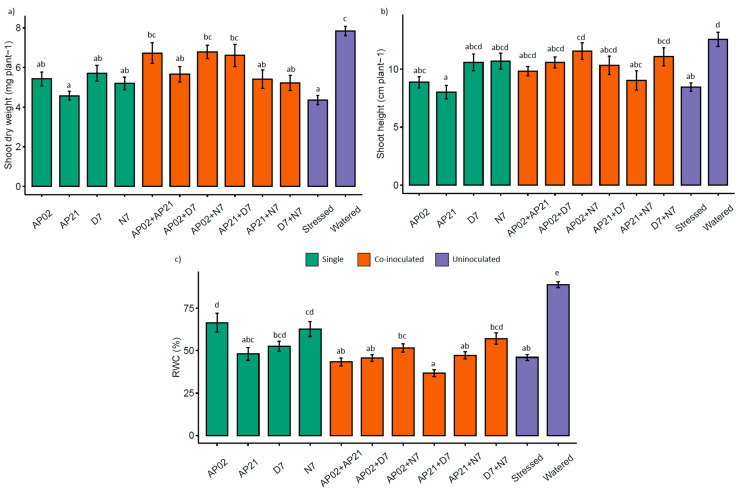
Response of perennial ryegrass plants after 10 days without irrigation. (**a**) Shoot dry biomass, (**b**) shoot height, and (**c**) relative water content (RWC). Bars represent the standard deviation of nine replicates. Different letters indicate that groups are significantly different according to the Kruskal–Wallis and pairwise comparison (*p* < 0.05). AP02: *Herbaspirillum* sp.; AP21: *Herbaspirillum* sp.; D7: *Azospirillum brasilense*; N7: *Pseudomonas fluorescens.*

Evaluation under controlled conditions revealed that water deficit significantly decreased the shoot biomass, height, and RWC (Figure 2). Compared with the watered control plants, the shoot dry biomass was reduced by about 50% in the stressed control plants. Moreover, RWC of the leaves reached 40% in the stressed control plants. The inoculation of beneficial bacteria (single as well as co-inoculation) had a significant effect on the shoot dry biomass, height, and RWC under water deficit (*p* < 0.01). Compared with the stressed control, two single strains, namely *Herbaspirillum* sp. AP02 and *P. fluorescens* N7, were able to improve RWC of the leaves by 20%. Moreover, three co-inoculation combinations, namely, (1) *Herbaspirillum* sp. AP02 and *Herbaspirillum* sp. AP21, (2) *Herbaspirillum* sp. AP02 and *P. fluorescens* N7, and (3) *Herbaspirillum* sp. AP21 and *A. brasilense* D7, increased the dry biomass production by 54.3%, 55.7%, and 51.6%, respectively. Given the agronomical importance of biomass production, these three co-inoculation combinations were selected for further experiments.

### 3.3. In Vitro PGP Traits of the Bacteria in the Selected Co-Inoculation Combinations

In vitro assessment revealed the individual and combined capacities of the four strains to produce ICs and EPSs and to promote plant growth without any contact with their host (via the production of VOCs) (Table 2). Regarding the behavior of the individual strains, *Azospirillum* sp. D7 showed the highest capacity to produce both ICs and EPSs. This strain produced six- and tenfold higher quantities of ICs and EPSs, respectively, than P. *fluorescens* N7, which showed the lowest capacity to produce both ICs and EPSs.

**Figure 3:**
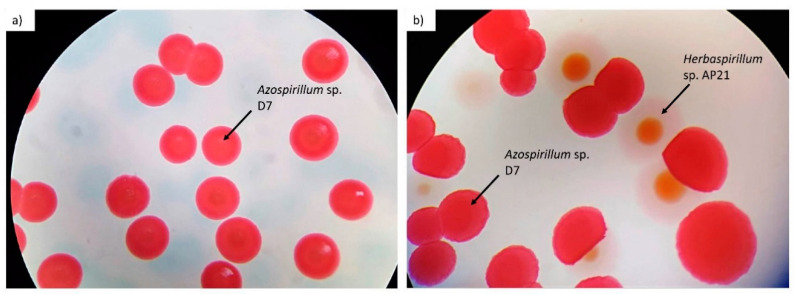
Colonies observed in the *Herbaspirillum* sp. AP21 and *Azospirillum* sp. D7 co-inoculations for the (**a**) indolic compounds and (**b**) exopolysaccharide determinations in congo red medium after 48 h of incubation at 30 °C. The colonies were observed in a stereoscopic microscope (Olympus, SZX7, Shinjuku, Japan) with a 0.8× zoom.

The production of ICs or EPSs by the two *Herbaspirillum* strains (AP02 and AP21) did not differ significantly under individual inoculation and co-inoculation as both strains behaved similarly. Moreover, the production of these compounds by co-inoculated *Herbaspirillum* sp. AP02 and P. *fluorescens* N7 was very similar to that by the individual *Pseudomonas* strain, which was confirmed by the cell count (CFU mL^−1^), wherein only *Pseudomonas* colonies were detected. However, IC production by co-inoculated *Herbaspirillum* sp. AP21 and A. *brasilense* D7 was higher than that by the individual strains, although only *Azospirillum*-like colonies were detected after the incubation period (Figure 3a). In the EPS assay, both *Herbaspirillum* sp. AP21 and A. *brasilense* D7 colonies were detected after 96 h of incubation (Figure 3b); however, the production was slightly lower than that by A. *brasilense* D7.

In the VOC assay, the inoculation of endophytic bacteria showed no significant effect on seed germination; however, the radicle length significantly increased (*p* < 0.05) with the individual inoculation and co-inoculation of most strains. Compared with the uninoculated control, the individual inoculation of P. *fluorescens* N7 and inoculation of *Herbaspirillum* sp. AP02 and *Herbaspirillum* sp. AP21 led to a 77% and 61% increase, respectively, in the radicle length (Table 3).

### 3.4. Plant Tissue Colonization

Endophytic bacteria that could grow in nitrogen-free medium were present in the inoculated and uninoculated seedlings (Table 4). In the uninoculated control, the concentration of these bacteria reached 10^3^ CFU g^−1^ of plant tissue; however, when the strains were individually inoculated or co-inoculated in the seeds, the bacterial concentrations in the shoots and roots of 7-day-old seedlings increased and reached approximately 10^5^ and 10^6^ CFU g^−1^, respectively, suggesting the ability of these strains to colonize and mobilize inside their host.

## 4. Discussion

Perennial ryegrass is widely used for livestock feeding in temperate regions, particularly in areas where good water supply can be ensured [49]. Unfortunately, because of climate change events, water resources are becoming scarce and rainfalls are becoming increasingly unpredictable, threatening the production of this temperate grass [24]. The present study aimed to explore the potential of the co-inoculation of four strains of endophytic PGPB to promote the growth of perennial ryegrass under water deficit.

We explored the taxonomy of these strains based on complete genome analysis, which can overcome the limitations of 16S rRNA to differentiate microorganisms at the species level and obtained two main results. The first result was the relatedness of the D7 strain to *A. brasilense.* In the case of *Azospirillum* strains, differences at the species level can lead to important differences in their interaction with their host and their PGP traits, including the pathways for IAA production and the ACC deaminase enzyme, as demonstrated by Wisniewski-Dyé et al. [45]. The second result was the relatedness of the N7 strain to *P. fluorescens* F113. The F113 genome has been reported by Redondo-Nieto et al. [50], who have highlighted the outstanding rhizocompetence of the strain and suggested that it could be related to the presence of 344 genes in this *Pseudomonas* strain that are not present in its closest relatives. This information could help us understand the potential of the N7 strain and target specific traits based on their phylogeny in future studies. For the *Herbaspirillum* strains AP02 and AP21, further molecular studies are needed as we evidenced that the strains belonged to different species than the ones used in the phylogeny tree.

The evaluation of water deficit under controlled conditions revealed that the dry biomass of the plants increased when some of these strains were co-inoculated, indicating that a possible interaction between these strains had a beneficial effect on the plants. Nevertheless, some combinations of the strains resulted in a reduction in the growth-promoting effect of the individual strains. Some authors have also observed this behavior and have concluded that the competition between bacteria could alter their ability to colonize the rhizosphere. Lucas García et al. [51] found that the sequence of the inoculation of bacterial strains altered the growth-promoting effect of *Pseudomonas fluorescens*, *Chryseobacterium balustinum*, *Serratia fonticola*, and *Sinorhizobium fredii*. In their study, inoculating the *S. fredii* strain first and the other strains five days later resulted in a synergistic effect on plant growth, which was not observed by co-inoculating the strains at the same time. This alteration of the ability to colonize the host might be due to the interruption in quorum sensing processes caused by the presence of different bacteria in mixed inoculants [52]. Because of the importance of quorum sensing for biofilm formation and persistence in the environment, the ability of some bacteria to interrupt these processes (by producing enzymes that destroy autoinducer signal molecules) has been utilized as a biocontrol mechanism against pathogenic bacteria [53], but this can also occur between beneficial microbes. Other antimicrobial compounds can also affect the interactions of different bacteria. Maroniche et al. [54] found a strong inhibitory effect of *Pseudomonas* strains over *Azospirillum brasilense* cells and were able to identify the production of siderophores, the type VI secretion systems, and the production of antibiotics as responsible for this effect. This was also observed in vivo, as the combination of these strains resulted in less viable cells of genus *Azospirillum* associated with wheat roots. This highlights the importance of testing different consortia in every plant host, in order to successfully engineer a consortium that could have a synergistic effect on plant growth and not a deleterious one.

In our study, we observed the synergistic effect with three PGPB consortia. Successful PGPB consortia have also been reported previously. Kumar et al. [55] observed a synergistic effect of the co-inoculation of *P. putida* NBRIRA and *Bacillus amyloliquefaciens* NBRISN13 in chickpea (*Cicer arietinum* L.) plants after 15 days without irrigation. They found that the consortium increased the biomass production and reduced the activity of enzymes related to ROS-scavenging systems. Moreover, Kakar et al. [56] found that a consortium of *B. amyloliquefaciens* Bk7 and *Brevibacillus laterosporus* B4 could mitigate the effect of 16 days of water deficit in rice (*Oryza sativa* L.) plants, improving their survival rate and reducing the leaf damage caused by water deficit. The authors observed the ability of the strains to modify the expression of genes related to stress response. Although some studies have used consortia of PGPB, there is no available information on the effect of *Azospirillum, Herbaspirillum*, and/or *Pseudomonas* consortia on plant growth under water deficit. Our results showed that these consortia have the potential to mitigate the effect of this stress in perennial ryegrass.

The increase in biomass production under water deficit could be explained by the ability of the strains to produce phytohormones, such as IC, cytokinins, and abscisic acid; to produce EPS; to reduce the plant’s ethylene levels with the ACC deaminase enzyme; and to induce systemic tolerance [57]. Of these mechanisms, the production of IAA is one of the most reported mechanisms for plant growth promotion under a diverse set of environmental conditions [58]. The production of ICs plays a major role in stress mitigation by modifying root traits, thereby widening the area for exploration and water uptake [59]. In the present study, the strains belonging to the genera *Azospirillum* and *Herbaspirillum* showed a high capacity to produce these compounds, and this capacity increased when *A. brasilense* D7 and *Herbaspirillum* sp. AP21 were co-inoculated. *Azospirillum* strains have been reported to have a superior capacity to produce ICs compared with *Herbaspirillum* strains [60,61]. Moreover, the yield per bacterial population of *A. brasilense* D7 and the two *Herbaspirillum* strains (AP02 and AP21) was very similar. In *Azospirillum,* the production of these compounds has been acknowledged as a key PGP trait, mainly via tryptophan-dependent pathways [62]. We observed the presence of *ipdC* in both *A. brasilense* D7 and *Herbaspirillum* sp. AP02, but not in *Herbaspirillum* sp. AP21.

When *Herbaspirillum* sp. AP21 and *A. brasilense* D7 were co-inoculated, we were only able to detect *Azospirillum* colonies. This result could be due to the role of ICs in intercellular interactions in microbial communities. ICs are involved in diverse microbial interactions, such as biofilm formation, motility, virulence, plasmid stability, and antibiotic resistance [63]. Somers et al. [64] revealed that *A. brasilense* can produce phenylacetic acid, an auxin-like antimicrobial compound, and suggested that this compound can play a major role in the inhibitory effect against other gram-negative bacteria. More studies are required to determine the kinetics of both the strains in the medium in order to understand why there was an increase in the production of these compounds and which strain was responsible for the production.

The strains used in the present study also produced EPSs. EPS production is one of the most important traits when screening for beneficial bacteria for promoting growth under abiotic stress conditions. The presence of EPSs guarantees a protective environment for bacterial cells against desiccation and is key for microbial aggregation, biofilm formation, and attachment to plant roots [65]. In the present study, *A. brasilense* D7 showed the highest ability to produce EPSs. This ability has already been proven to be necessary for plant–*Azospirillum* interactions [66,67] and has been shown to protect *Azospirillum* cells from high and low temperatures, extreme pH, and drying [68]. In plant–*Azospirillum* interactions, EPSs have been found to improve plant growth under low nitrogen conditions [69,70] and drought [71,72]. When *Herbaspirillum* and *Azospirillum* strains were co-inoculated, both the bacteria were able to co-exist for 96 h, which opens an opportunity for preparing a possible consortium formulation of these strains.

Although EPS production is required for biofilm formation in *Pseudomonas* strains [73], the production of this compound was the lowest under the evaluated conditions. However, the *Pseudomonas* strain showed the ability to promote growth in the VOC assay. VOCs have recently received great importance, given the growing evidence of their ability to modulate plant growth, development, and defense and to act as a signal communication at the microorganism level and plant–microorganism level [74]. The ability of *Pseudomonas* to produce VOCs and promote plant growth has been proven in tomato [75] and tobacco plantlets [42]; however, most research has focused on their biological control effect [76,77,78]. There is not much information about the ability of *Azospirillum* and *Herbaspirillum* to produce VOCs; however, our results suggest that they are also able to produce volatile compounds that exert an effect on radicle length.

In summary, we aimed to gather information about the synergy between co-inoculation treatments using four PGPB to promote the growth of ryegrass plants under water deficit. We observed that these bacteria have the metabolic battery to colonize plant tissue, produce ICs and EPSs, and promote the growth of their host by producing VOCs. These results open an opportunity to continue studying the plant–microbe relationship. Future studies should evaluate the physiological and biochemical responses of plants to water deficit with a special focus on the *Herbaspirillum* and *Azospirillum* consortium in order to help understand the effects of this co-inoculation on the response of perennial ryegrass to water deficit.

## 5. Conclusions

The endophytic bacteria used in the present study showed different mechanisms for promoting plant growth under water deficit. However, we found that the *A. brasilense* D7 and *Herbaspirillum* sp. AP21 consortium has potential and should be further studied because of its ability to co-exist and to produce higher amounts of ICs together and, most importantly, to promote dry biomass production in perennial ryegrass under water deficit.

## Figures and Tables

**Table 1:** Representative genes selected to explore the potential growth-promoting traits of the bacteria. AP02: *Herbaspirillum* sp.; AP21: *Herbaspirillum* sp.; D7: *Azospirillum brasilense*.; N7: *Pseudomonas fluorescens*. PGP, plant growth-promoting; ACC, 1-aminocyclopropane-1-carboxylate.

PGP Trait	Gene	Protein	AP02	AP21	D7	N7	Reference
ACC deaminase	*acdS*	1-aminocyclopropane-1-carboxylate deaminase	+	+	−	+	Glick et al. [44]
Indolic compounds production	*iaaM*	Monoamine oxidase	-	-	-	+	Wisniewski-Dyé et al. [45]
	*ipdC*	Indole-3-pyruvate decarboxylase	+	-	+	-	Wisniewski-Dyé et al. [45]
Exopolysaccharides (EPS) and Lypopolysaccharides production (LPS)	*noeJ*	Mannose-1-phosphate guanylyltransferase	-	-	-	-	Lerner et al. [46]
	*noeL*	GDP-mannose 4,6-dehydratase	-	-	-	-	Lerner et al. [46]
	*rfbB*	dTDP-glucose 4,6-dehydratase	-	-	+	+	Balsanelli et al. [43]
	*rfbC*	dTDP-4-dehydrorhamnose 3,5-epimerase	-	-	+	-	Balsanelli et al. [43]
Colonization: Biofilm formation	*flmA*	Putative sugar nucleotide epimerase/dehydratase	-	-	+	-	Rossi et al. [47]
	*flmB*	Flagellin modification protein FlmB	-	-	+	-	Rossi et al. [47]
Volatile Organic Compounds	*gacA*	Response regulator GacA	-	-	-	+	Cheng et al. [48]
	*gacS*	Histidine kinase	-	-	-	+	Cheng et al. [48]

**Table 2:** Plant growth-promotion activities of individual and co-inoculated strains. The data were analyzed by Kruskal–Wallis, and a Wilcoxon test (*p* < 0.05) was performed to compare group means against the non-inoculated stressed treatment. The strains AP02 and AP21 correspond to the *Herbaspirillum* genus, and D7 and N7 to *Azospirillum* and *Pseudomonas*, respectively. CFU, colony forming units.

Treatment	Indolic Compounds		
	μg mL^−1^	μg LogCFU^−1^	Morphology Observed
AP02	42.9 ± 3.0 b	13.5 ± 1.1 bc	*Herbaspirillum*
AP21	45.5 ± 1.8 b	11.9 ± 0.5 b	*Herbaspirillum*
D7	69.7 ± 1.0 c	12.4 ± 1.3 bc	*Azospirillum*
N7	11.9 ± 1.6 a	1.7 ± 0.2 a	*Pseudomonas*
AP02 + AP21	39.7 ± 0.3 b	7.5 ± 0.3 b	*Herbaspirillum*
AP02 + N7	14.8 ± 3.7 a	2.3 ± 0.5 a	*Pseudomonas*
AP21 + D7	90.8 ± 3.9 d	14.5 ± 1.0 c	*Azospirillum*
**Treatment**	**Exopolysaccharides**		
	**mg mL^−1^**	**mg LogCFU^−1^**	**Morphology Observed**
AP02	4.8 ± 0.5 b	1.0 ± 0.1 b	*Herbaspirillum*
AP21	5.4 ± 0.6 b	1.0 ± 0.1 b	*Herbaspirillum*
D7	24.5 ± 1.0 d	3.7 ± 0.3 d	*Azospirillum*
N7	2.4 ± 0.7 a	0.3 ± 0.0 a	*Pseudomonas*
AP02 + AP21	4.6 ± 1.0 b	0.8 ± 0.2 b	*Herbaspirillum*
AP02 + N7	2.6 ± 0.3 a	0.3 ± 0.0 a	*Pseudomonas*
AP21 + D7	14.9 ± 1.1 c	3.0 ± 0.2 c	*Azospirillum* + *Herbaspirillum*

Data are presented as mean ± standard deviations. Means and standard deviations are the results of three replicates. Different letters represent statistically different groups according to the Kruskal–Wallis and pairwise comparison (*p* < 0.05).

**Table 3:** Effect of volatile organic compounds (VOCs) of individual strains and co-inoculations of endophytic bacteria on seed germination, coleoptile, and radicle length of perennial ryegrass. The data were analyzed by Kruskal–Wallis, and a Wilcoxon test (*p* < 0.05) was performed to compare group means. The strains AP02 and AP21 correspond to the *Herbaspirillum* genus, and D7 and N7 to *Azospirillum* and *Pseudomonas*, respectively.

Treatment	Seed Germination	Length (mm)	
	(%)	Coleoptile	Radicle
Uninoculated	70.0 ± 5.7 a	4.6 ± 1.3 a	8.2 ± 0.6 a
AP02	67.5 ± 4.2 a	6.0 ± 1.3 a	12.0 ± 1.1 ab
AP21	78.3± 8.4 a	4.6 ± 0.5 a	10.3 ± 1.7ab
D7	74.1 ± 15.7 a	6.0 ± 0.7 a	12.4 ± 1.3 ab
N7	63.3 ± 11.5 a	7.4 ± 1.9 a	14.5 ± 1.8 b
AP02 + AP21	62.5 ± 6.8 a	6.2 ± 1.2 a	13.2 ± 2.5 b
AP02 + N7	63.3 ± 14.1 a	5.0 ± 0.6 a	11.2 ± 1.8 ab
AP21 + D7	65.8 ± 2.6 a	5.2 ± 1.8 a	11.7 ± 0.7 ab

Data are presented as mean ± standard deviations. Means and standard deviations are the results of four replicates. Different letters represent statistically different groups according to the Kruskal–Wallis and pairwise comparison (*p* < 0.05).

**Table 4:** Bacterial cell count in semi-solid nitrogen-free medium (NFb) in seven days old ryegrass seedlings.

Treatment	Cell Count (CFU g^−1^)	
	Shoot	Root
Uninoculated	2.5 × 10^3^	4.0 × 10^2^
AP02	7.5 × 10^5^	7.0 × 10^5^
AP21	9.5 × 10^5^	2.0 × 10^7^
D7	1.1 × 10^7^	1.5 × 10^2^
N7	1.5 × 10^9^	7.0 × 10^6^
AP21 + D7	4.5 × 10^6^	4.5 × 10^4^
AP02 + D7	4.5 × 10^6^	4.5 × 10^6^
AP02 + N7	7.0 × 10^6^	1.5 × 10^7^

## Data Availability

Data is contained within the article.

## Abbreviations

ACC	1-aminocyclopropane-1-carboxylate
CFU	Colony forming units
DW	Dry weight
EPS	Exopolysaccharide
FW	Fresh weight
IAA	Indole-3-acetic acid
IC	Indolic compounds
LPS	Lypopolysaccharides
PGPB	Plant growth-promoting bacteria
RWC	Relative water content
TW	Turgid weight
VOC	Volatile organic compound
